# Experiences of neurofeedback therapists in treating attention-deficit hyperactivity disorder

**DOI:** 10.4102/hsag.v27i0.1874

**Published:** 2022-07-28

**Authors:** Jessica Oliveira, Janice Pellow, Tebogo Tsele-Tebakang, Elizabeth M. Solomon

**Affiliations:** 1 Department of Complementary Medicine, Faculty of Health Sciences, University of Johannesburg, Johannesburg, South Africa

**Keywords:** neurofeedback therapist, neurofeedback, experiences, attention-deficit hyperactivity disorder, qualitative, phenomenological

## Abstract

**Background:**

Neurofeedback is a neurostimulatory biofeedback behavioural therapy that regulates brain wave levels for optimal cognitive functioning. It has shown promising results for the treatment of attention-deficit hyperactivity disorder (ADHD).

**Aim:**

To explore and describe the experiences of neurofeedback therapists (NTs) using neurofeedback to treat children and adults with ADHD and their experience of its role and value in treating this condition.

**Setting:**

Interviews were conducted in participants’ private consultation rooms in Gauteng, South Africa.

**Methods:**

In this qualitative study using a descriptive phenomenological approach, six registered NTs with experience of treating ADHD were interviewed. Participants were asked: ‘Tell me about your experiences of neurofeedback with ADHD patients in your practice.’ Responses were audio-recorded, transcribed and underwent thematic analysis.

**Results:**

Participants experienced neurofeedback as an effective treatment for ADHD and other coexisting conditions, such as anxiety and insomnia. Participants felt that the neurofeedback process facilitates a beneficial therapeutic relationship and integrates well with other treatment methods. Challenges faced in practice included certain underlying comorbidities, home environment, poor patient compliance and NTs’ level of expertise, which impact treatment outcomes.

**Conclusion:**

Neurofeedback therapists expressed an overall positive perception of the value of neurofeedback to reduce ADHD symptom severity and improve quality of life, particularly when used as part of a multimodal approach. Participants identified a need for further education and awareness regarding the use of neurofeedback for ADHD.

**Contribution:**

This study has contributed to our understanding of the role of neurofeedback in treating ADHD in the South African setting.

## Introduction

Attention-deficit hyperactivity disorder (ADHD) is an increasingly prevalent childhood neuropsychiatric disorder that often persists into adulthood (Schoeman & Liebenberg 2017). Attention-deficit hyperactivity disorder has a worldwide pooled prevalence of 5.29% in children up to the age of 18 years (Polanczyk et al. 2007) and between 2.58% and 6.76% in the adult population (Song et al. 2021). Whilst accurate prevalence statistics for South Africa are lacking, ADHD is estimated to affect at least 5% of children and around 1.09% of adults (Schoeman & Liebenberg 2017; Schellack et al. 2016). This disorder is more common in males than in females, with a ratio in community and clinical samples ranging from 2.4:1 to 4.0:1 (Vuori et al. 2020). Non-pharmacological treatment strategies for ADHD include behavioural and environmental modifications, whilst pharmacological therapies primarily involve the use of stimulant and non-stimulant medications. Although pharmacotherapy plays an important therapeutic role, ADHD is a complex condition and therefore appropriate treatment plans should be tailored to the individual in a comprehensive multidisciplinary management approach (Brown, Samuel & Patel 2018).

Neurofeedback is a type of biofeedback therapy that involves a real-time brain–computer interface, usually using an electroencephalogram; the feedback loop that is created allows the individual to learn to normalise their brain activity by directly altering the underlying neural mechanisms of cognition and behaviour (Enriquez-Geppert et al. 2019). Attention-deficit hyperactivity disorder sufferers have a higher incidence of aberrant brain waves, with predominantly elevated levels of theta waves, lower sensorimotor rhythms and low levels of beta waves, thereby affecting attention, memory processes and movement control. Neurofeedback training allows the patient to learn how to maintain appropriate levels of these brain waves for optimal functioning (Marzbani, Marateb & Mansourian 2016). Numerous clinical trials have found that neurofeedback improves attention levels and behaviour and decreases hyperactivity in ADHD (Baena et al. 2021). Meta-analyses have confirmed the significant efficacy of neurofeedback for ADHD, with a medium effect size and 32% – 47% remission rates, which demonstrated sustained effects when assessed after 6–12 months (Arns et al. 2020). Neurofeedback is also used in neuropsychological disorders such as anxiety and depression (Omejc et al. 2019), which are potential comorbidities of ADHD (Panevska et al. 2015).

### Problem statement

Attention-deficit hyperactivity disorder is a chronic and complex disorder that comes with a chain of difficulties, including impairments in academic, social and neuropsychological functioning (Mayer et al. 2015). Pharmacological therapy remains the primary form of treatment but is not without the risk of adverse effects. As a result, individuals struggling with ADHD may seek out alternative forms of treatment to manage their symptoms (Catalá-López et al. 2017). Neurofeedback is a non-invasive and non-pharmacological therapy that offers a promising adjunctive approach to manage ADHD in both children and adults (Riesco-Matías et al. 2021); however, its use in the South African setting is limited. There have been no studies to date regarding the experiences of South African neurofeedback therapists (NTs) in the use of neurofeedback to treat this condition.

### Purpose of the study

The purpose of the study was to explore and describe the experiences of NTs using neurofeedback to treat children and adults with ADHD and their experience of its role and value in treating this condition.

## Research methodology

### Study design

A qualitative, descriptive phenomenological design was chosen for this study. Phenomenology is the philosophical study of conscious experience as experienced from the subjective viewpoint and involves exploring the ‘lived experiences’ of the participants. Descriptive phenomenology focuses on describing a phenomenon from the perspective of those who have experienced it. It is considered a useful research approach that is well suited to assist one in learning from the experiences of others (Sundler et al. 2019; Tenny et al. 2021).

### Study setting

This study was conducted in the private consultation rooms of participative NTs in Gauteng province, South Africa.

### Population and sampling

The research population included NTs practising in South Africa. Participants were recruited by means of purposive sampling. The contact details of registered NTs are freely available on the Biofeedback Association of South Africa (BFSA) website. When the study was initiated, there were 20 registered neurofeedback practitioners in South Africa situated in Gauteng, KwaZulu-Natal, Western Cape and Northwest provinces, with most of them being located in Gauteng. To practise as an NT, healthcare professionals must be trained in neurofeedback and registered with the Health Professions Council of South Africa, the Allied Health Professions Council of South Africa, the Nursing Council or the Social Workers Professional Board and the Biofeedback Certification International Alliance (BCIA) (BFSA, n.d.). The study’s inclusion criteria were as follows: NTs registered with the BCIA and their respective professional council, practising in South Africa, who use neurofeedback as their primary form of treatment for ADHD and have treated a minimum of 10 ADHD patients prior to the interview. Participants were interviewed until data saturation was reached, which is widely used to determine the sufficiency of sample size in qualitative research, when no new data or themes emerge (Vasileiou et al. 2018). Six NTs from Gauteng participated in the study.

### Data collection procedures

Individual, semi-structured, in-person interviews were conducted using open-ended questions for greater flexibility and to allow participants to freely describe their experiences (Tanwir, Moideen & Habib 2021). Participants were asked a central question: ‘Tell me about your experiences of neurofeedback with ADHD patients in your practice.’ This was followed up with probing sub-questions to facilitate the interview process and allow for better understanding and clarification:

- In your experience, what is the role of neurofeedback in treating ADHD?
- How long on average before an effect is noticed with neurofeedback in your ADHD patients?
- Do you find neurofeedback to be a valuable tool for ADHD patients in your practice?
- What are the challenges you face when dealing with ADHD patients in particular?
- What challenges do you experience with neurofeedback as a therapy?

Interviews were conducted privately in participants’ consultation rooms, at their convenience, and were audio-recorded, lasting an average of 30 min. Bracketing was used to minimise the researcher bias. Bracketing is the process of setting aside existing perceptions of a particular phenomenon to receive new information in an open, non-judgemental way (Sundler et al. 2019). The researcher critically reflected on her own pre-understanding of the phenomenon and was assisted in this, by using reflective journaling and field notes.

### Thematic analysis

Interviews were transcribed by the researcher and thematic analysis was used to analyse the data. According to Sundler et al. (2019), thematic analysis can be applied to phenomenological research to derive meaning-oriented themes from an understanding of patterns of meaning from participants’ lived experiences. They describe the three-stage process of analysis used in this study. In the first stage, the researcher became familiar with the data by reading and exploring its meaning with open-mindedness. The second stage involved searching for in-depth meanings in the data; these were marked, notes were made and then coloured codes were assigned. These identified coded meanings were compared and organised into patterns, from which themes emerged. Qualitative analysis software Atlas.ti version 8 was used to assist this process. In the third stage, themes were arranged into a ‘meaningful wholeness’. Themes were given explicit names that best described the lived experiences in its context.

### Measures of trustworthiness

Trustworthiness was ensured through credibility, transferability, dependability and confirmability, which enhance the rigour of the study (Korstjens & Moser 2018). To ensure credibility, the methodological procedures and analytical methods are presented as thoroughly and transparently as possible. The themes and subthemes are described and supported by participants’ verbatim quotes. Triangulation methods employed include individual interviews until data saturation was reached, and an extensive literature control compiled on the phenomenon. Participants were encouraged to express themselves openly, and each interview was audio-recorded to enhance accuracy. The transcription of recordings was undertaken by the researcher herself, which enhanced familiarity with the content, and qualitative analysis software was used to facilitate the analysis. Data analysis was verified by an independent qualitative analyst, which further ensured credibility. Transferability was enhanced by using purposive sampling to select information-rich participants. Dependability was facilitated by providing detailed documentation of the methods used, which would allow others to duplicate the study. A pilot study was undertaken to verify and refine the central and probing sub-questions posed to participants. Reflexivity involves the researcher’s capacity for self-reflection (Korstjens & Moser 2018) and contributes to the confirmability of the study. This process was enhanced through researcher bracketing, use of field notes and memos in the coding process. A confirmatory audit trail was created in terms of transcriptions, lists of codes, notes and data analysis.

### Ethical considerations

Permission to conduct the research was obtained from the University of Johannesburg Faculty of Health Sciences’ Research Ethics Committee, (ethical clearance number: REC-241112-035). Participants were provided an information letter detailing the study’s purpose and procedures; signed consent forms were obtained from the participants to participate in the study and for the interview to be audio-recorded. Participation was confidential and participants’ names were replaced with numbers and no identifying detail was linked to the data presented. Participants were treated with respect and their right to autonomy was respected. Participation was voluntary in that they could withdraw from the study at any time without repercussion.

## Results

### Demographic profile of participants

Six NTs (one man and five women) were interviewed, and participants had between 3 and 19 years of experience using neurofeedback in their practices (Table 1).

**TABLE 1:** The relationship between the number of years in practice and the number of years using neurofeedback, as well as the profession of each participant.

Participant Number	Number of years in practice	Number of years using neurofeedback	Profession
Participant 1	17	15	Homeopath
Participant 2	22	19	Educational psychologist, psychometrist
Participant 3	7	6	Counsellor
Participant 4	7	7	Psychologist
Participant 5	3	3	Psychometrist
Participant 6	17	16	Counsellor

*Source*: Oliveira, J., 2019, ‘Neurotherapists’ experiences of the use of neurofeedback in the treatment of attention deficit and hyperactivity disorder’, Masters Dissertation, University of Johannesburg, Johannesburg, viewed 15 November 2021, from http://hdl.handle.net/10210/412946.

## Findings and discussion

Thematic analysis revealed three themes and their associated subthemes. The themes identified are (1) experiences with using neurofeedback for ADHD, (2) its value to the practice and (3) public perceptions. These are presented and supported by participants’ verbatim quotations and are discussed in terms of literature control. Participant identifiers for each quote consists of the participant number, number of years using neurofeedback, and occupation in brackets.

### Theme 1: Experiences with using neurofeedback for attention-deficit hyperactivity disorder

Participants detailed their experience of using neurofeedback for ADHD and indicated it as an effective treatment method, and its effectiveness can be determined through general and specific symptom improvement that is observable within a few months of treatment. However, they acknowledge that neurofeedback treatment is not without limitations and challenges.

#### Subtheme 1: An effective treatment method for attention-deficit hyperactivity disorder

Participants in the study were from varied professions (Table 1), but experienced neurofeedback as being very effective and the preferred treatment modality for ADHD:

‘So, I see a lot of ADHD kids and adults, with very good results.’ (Participant 3, 6 years, Counsellor)

‘I think neurofeedback should be the first option for treatment (for ADHD).’ (Participant 1, 15 years, Homeopath)

Participants determined the effectiveness of treatment by reported patient improvements in general well-being, such as sleep, as well as ADHD and associated symptoms, such as cognitive function, anxiety and hyperactivity:

‘[…*P*]arents first notice better sleep, improvements in handwriting and then the other symptoms, such as getting better generally.’ (Participant 1, 15 years, Homeopath)

‘But most of [*the*] time there is a significant improvement in concentration, anxiety, processing ability and in terms of sleep.’ (Participant 6, 16 years, Counsellor)

Neurofeedback is effective because it addresses the patient holistically and individually, which is evident in following participant’s quotes:

‘[… *T*]hat is why it is effective, because we are not working with one blanket diagnosis, we are working with how this specific dysregulation affects that specific person.’ (Participant 6, 16 years, Counsellor)

Participants agreed that the improvement of symptoms is observable within the first few months of treatment:

‘As a therapy modality it’s something that’s great … because you do see the results fairly quickly.’ (Participant 1, 15 years, Homeopath)

‘On average, from session three or four most people start seeing an improvement.’ (Participant 4, 7 years, Psychologist)

The benefits of neurofeedback therapy extend beyond the treatment period, according to some participants, as it provides their patients with coping mechanisms:

‘[… *S*]o usually patients will still see an improvement even after the neurofeedback sessions have ceased.’ (Participant 2, 19 years, Educational psychologist, Psychometrist)

‘It enables our patients to regulate themselves so that they will always have that long-term coping mechanism they can bring home.’ (Participant 4, 7 years, Psychologist)

According to a systematic review by Baena et al. (2021), several clinical studies have revealed the many benefits of neurofeedback for patients with ADHD, in producing positive and long-lasting effects on behaviour, attention, hyperactivity and impulsivity. Additionally, it has also been shown to improve motor control and coordination. Patients with ADHD often exhibit poor motor control and have coordination difficulties, which in turn can impact the performance of tasks, such as writing, and leads to poor academic performance (Singh et al. 2015). A systematic review and meta-analysis by Arns et al. (2020) confirmed a therapeutic effect for at least 6 months after treatment.

#### Subtheme 2: Limitations and challenges

Participants acknowledged limitations to neurofeedback treatment and identified challenges that limit its effectiveness. These include certain comorbidities, a dysfunctional home environment, lack of patient compliance and feedback and the experience level of the NT, which are presented individually.

**Comorbidities:** Participants experienced that the severity of the condition and certain underlying physiological and psychological comorbidities can result in patients not being amenable to treatment:

‘[… *I*]f there is a physiological issue that is causing the ADHD, neurofeedback is limited in that it cannot cure that issue.’ (Participant 1, 15 years, Homeopath)

‘[… *N*]eurofeedback doesn’t cure autism and doesn’t most often cure a hundred percent improvement [*sic*] in ADHD.’ (Participant 6, 16 years, Counsellor)

‘In terms of success with ADHD, it depends on the severity of the condition.’ (Participant 6, 16 years, Counsellor)

Whilst there is conflicting evidence for the use of neurofeedback for learning disabilities (e.g. dyslexia) and other neurodevelopmental conditions such as autism spectrum disorder, some studies show that specific types of neurofeedback may in fact be helpful (Datko, Pineda & Müller 2018). The differences in findings may show that the limitations of neurofeedback may be case-dependent or NT-dependent and not necessarily based on the underlying condition.

**Dysfunctional home environment:** Some participants stated that if problems arise from within the home and family, it can impede patient improvement, or the treatment may not be successful:

‘I’m not a psychologist or a counsellor so if there are external factors … those sorts of things can almost put a person back in terms of progression. So, the patient may have some type of improvement, but their parents are going through a divorce … neurofeedback cannot help in that aspect.’ (Participant 1, 15 years, Homeopath)

‘If that person’s family system is in dysfunction [*sic*], that patient is totally dysfunctional. No amount of therapy or medication is going to sort that out.’ (Participant 6, 16 years, Counsellor)

Studies have confirmed a link between negative family situations and severe ADHD symptoms in children (Hawes et al. 2013). A longitudinal study sampling 197 3-year-old preschoolers showed that family dysfunction can contribute to the worsening of ADHD symptoms in children (Breaux & Harvey 2019).

**Lack of feedback:** Participants experienced a lack of feedback from some parents or caregivers, who do not communicate with the NT, which limits their ability to assess patient progress:

‘We do get parents we never see … so we struggle in terms of feedback from parents.’ (Participant 3, 6 years, Counsellor)

‘Another thing is feedback from the parents during the treatment. Sometimes you get parents who just drop off their kids and you never see them for some face-to-face feedback.’ (Participant 5, 3 years, Psychometrist)

**Poor compliance:** Participants experienced a lack of patient compliance, a challenge when treating ADHD, and all NTs experienced this as a substantial obstacle. They expressed that poor treatment compliance, in terms of missed or prematurely terminating treatments, causes restrictions in the treatment protocol and hinders patient improvement. They attributed the lack of compliance to the time commitment required, usually two sessions a week for 3–4 months, and the cost of treatment:

‘[… *What*] plays a role in patient compliance [*is*] whether they want to terminate the sessions before we [*are*] even finished, which is one of the drawbacks of this therapy.’ (Participant 3, 6 years, Counsellor)

‘A lot of people do not have the time to go through the therapy process; they want a quick fix and a miracle, so the amount [*sic*] of sessions is a bit of a drawback for some people.’ (Participant 4, 7 years, Psychologist)

‘Also, it can be quite expensive.’ (Participant 3, 6 years, Counsellor)

Neurofeedback is a time-consuming and cost-intensive therapy because of the number of sessions required, the expensive equipment needed to conduct the therapy and the labour required by the NT, who monitors the feedback in real time (Marzbani et al. 2016). Additionally, neurofeedback is not covered by most medical aids. This may be tremendously challenging for those with financial difficulties and limited time available to dedicate to this therapy. A study by Larson et al. (2012) also confirms that NTs experience poor compliance and attendance of sessions as a drawback to treatment.

**Neurofeedback therapist’s experience and expertise:** A participant expressed that NTs must be able to identify the most effective types of neurofeedback treatment for the patient and which one to avoid. The effectiveness of treatment is thus predicated based on the NT’s level of experience and expertise:

‘So, you need to know your patient. If you don’t take the time to know where each of these types of neurofeedback are [*sic*] going to cause problems for that person, you’re going to get a negative response.’ (Participant 6, 16 years, Counsellor)

‘As much as it is a wonderful tool to create improvement, if you change brain function … without knowing what you are doing, or what you are changing, it will create problems where there weren’t.’ (Participant 6, 16 years, Counsellor)

According to Han et al. (2016), the amount of direct involvement the NT has in the session can affect the outcome, as the feedback needs to be analysed live and in person, and each session is altered in line with the patient’s progress. There are various types of neurofeedback therapies, dependent on which brain activity is targeted. Slow cortical potential neurofeedback is often considered the preferred standard protocol for patients with ADHD and there is a growing body of evidence related to its effectiveness; however, several other protocols have also shown to have beneficial effects in terms of ADHD symptom reduction (Hasslinger et al. 2020). Therefore, therapeutic success is more likely if the NT is experienced and well trained.

### Theme 2: The value of neurofeedback in the practice

Participants experienced that using neurofeedback is beneficial in their practice as it enhances the therapeutic relationship with their patients and enables them to be part of an integrative multidisciplinary approach in treating ADHD.

#### Subtheme 1: The therapeutic relationship

Neurofeedback is patient orientated, which facilitates a therapeutic relationship that allows for open communication and encourages patients to be involved in their own healing, as explained by the following participants:

‘[… *Y*]ou establish a relationship and bond with them.’ (Participant 1, 15 years, Homeopath)

‘[… *Y*]ou show them that window into their brain … and how their brainwaves are going within that favoured threshold and they see that change and they start to see how powerful their brain really is … that is tremendously powerful in their progress of healing.’ (Participant 2, 19 years, Educational psychologist, Psychometrist)

The therapeutic relationship is an important aspect influencing treatment outcomes. Arnow and Steidtmann (2014) asserted that a healthy relationship allows for a collaborative effort between the patient and the healthcare provider in the attainment of specific treatment goals and is enhanced by the development of an emotional bond (e.g. caring and trust). According to Hasslinger et al. (2020), there is some debate whether the positive treatment effects of neurofeedback are because of the regulation of specific neurophysiological parameters in the brain or owing to non-specific factors related to the therapy, such as the positive therapeutic relationship with the NT, or the practice effects of sitting still for prolonged periods; however, positive reinforcement from a trusted NT enhances patient motivation, which in turn improves the efficacy of the treatment process.

#### Subtheme 2: Integrated multidisciplinary approach

Participants acknowledged the value of integrating neurofeedback into a multidisciplinary treatment approach involving other therapies to augment the treatment outcome. They refer patients when necessary and are part of a multidisciplinary team:

‘I work very closely with a group of varied practitioners [*who*] all understand the basics of neurofeedback, so that makes it ideal for them to treat the patient according to, let’s say their phenotypes and what supplements or homeopathic remedy will work for them.’ (Participant 2, 19 years, Educational psychologist, Psychometrist)

‘[… *B*]ut it’s something that works together with other interventions like supplementation, functional medicine and homeopathy.’ (Participant 1, 15 years, Homeopath)

‘So, if concentration is not a concern and only reading and writing [*is a problem*], maybe that patient doesn’t need neurofeedback but needs remedial lessons.’ (Participant 1, 15 years, Homeopath)

A systematic review by Razoki (2018) found that neurofeedback should be considered as an adjunctive therapy as part of a multimodal and multidisciplinary treatment approach for children and adolescents with ADHD, individualised to the needs of the patient. Baena et al. (2021) concluded that neurofeedback works well when it is combined with other interventions such as medication, physical activity, behavioural therapy training or attention training to improve ADHD symptoms in children.

Pimenta et al. (2021) revealed that neurofeedback produces sustained improvement and remission rates in almost half of all ADHD patients who use it and that combining neurofeedback with other interventions (specifically parental training, sleep hygiene and nutritional advice) appears to produce additive or synergistic effects.

### Theme 3: Public perceptions of neurofeedback

Most participants stated that the public’s perception of neurofeedback treatment impacts their practice and profession. Negative perceptions result in them needing to defend and convince others about the legitimacy and benefits of neurofeedback as a therapy, whilst some perceive this as an alternate treatment option for ADHD.

#### Subtheme 1: Negative perceptions

Participants experienced frustration over the negative reputation that neurofeedback has amongst some in the general public, educationists and healthcare providers, who are unfamiliar with neurofeedback. They attribute it to unfavourable reports caused by unqualified individuals practicing neurofeedback and the false, unrealistic advertising of its benefits:

‘There’s a lot of bad press out there regarding neurofeedback.’ (Participant 3, 6 years, Counsellor)

‘It is a very big challenge for us neurotherapists now because at the start there was a big group of practitioners who kind of sold neurofeedback as this cure-all therapy for every condition, which is a load of rubbish.’ (Participant 6, 16 years, Counsellor)

‘Those people aren’t even registered with the health professions council and it’s unfortunate because there is equipment that’s sold online.’ (Participant 6, 16 years, Counsellor)

A review by Renton, Tibbles and Topolovec-Vranic (2017) confirmed that there is an increasing number of unlicensed therapists practising as NTs and Larson, Ryan and Baerentzen (2010) concurred that negative opinions may also be the result of deceptive marketing, where unlicensed individuals market the therapy as a ‘cure all’. The negative perception of some professionals towards neurofeedback therapy is confirmed by Orndorff-Plunkett et al. (2017) who reported that whilst many healthcare practitioners support its use, some members of the medical and research communities exhibit mistrust and bias towards neurofeedback as a therapeutic option.

#### Subtheme 2: Convincing others

Most participants found that patients, parents and doctors need convincing of the benefits of neurofeedback for ADHD:

‘Even if people are coming here or bringing their children here and they are paying for it, they almost believe it’s not going to work, so you almost have to convince them the change they’re seeing is because of the neurofeedback.’ (Participant 3, 6 years, Counsellor)

‘I feel one of the biggest challenges is convincing doctors and parents that neurofeedback works.’ (Participant 4, 7 years, Psychologist)

This suggests the need for education on neurofeedback as a therapy for both the public and patients. According to Gold and McClung (2006), patient education significantly improves treatment compliance and enhances clinical benefits. Increasing the public’s awareness and understanding of neurofeedback remains a challenge.

#### Subtheme 3: An alternative approach

Participants indicated that many of their patients perceive neurofeedback therapy as a last line of treatment owing to dissatisfaction with conventional medication because of perceived ineffectiveness or adverse effects:

‘[… *O*]r it could be that they want to take them off of medication for whatever reason, like side effects.’ (Participant 1, 15 years, Homeopath)

‘[… *T*]heir medication is not working well enough.’ (Participant 1, 15 years, Homeopath)

‘[… *I*]t could be that they come to me because they don’t want to be on medication, so they don’t want to go that route.’ (Participant 1, 15 years, Homeopath)

Razoki (2018) reported that stimulant medications are widely used to treat ADHD; however, their limitations include a short duration of effectiveness, little or no response in some patients and short-term adverse effects such as poor appetite, nausea and fatigue. Furthermore, long-term adverse effects are poorly investigated. As such, patients may refuse or show poor compliance with drug treatment (Hasslinger et al. 2020). According to Thompson and Thompson (2016), there is also a level of dependence that may develop from using these medications. The use of complementary or alternative therapies is a growing trend amongst ADHD sufferers in the search for effective alternatives to conventional medications (Wang et al. 2020), but many complementary and alternative options lack sufficient research to support their use (Sarris et al. 2011).

Neurofeedback is shown to be useful for ADHD patients who do not respond to or who develop serious adverse effects to medication. Razoki (2018) also found neurofeedback as a possible alternative to stimulant medications for certain patients.

Conversely, the concomitant use of neurofeedback with medication may improve the effectiveness of the medication or allow for a reduction in dosage, which reduces the incidence of adverse effects (Razoki 2018; Baena et al. 2021).

### Limitations

Whilst NTs from the whole of South Africa, meeting the inclusion criteria, were invited to participate, only those from the Gauteng area agreed to do so. As some of NTs’ ADHD patients use an integrated approach to treatment, the specific effect of neurofeedback is difficult to determine in these cases. Other limitations are those inherent in a qualitative research approach, such as findings not being generalisable to the population. A limitation of neurofeedback as a treatment modality is the scarcity of registered NTs in South Africa and the time and costs involved mean the therapy, currently, is unavailable to the majority, or disadvantaged, ADHD sufferers.

### Recommendations

Strategies to educate the public on the benefits and scope of neurofeedback therapy, highlighting the NT’s educational and registration requirements, should be investigated. It is recommended that the experiences of patients receiving neurofeedback for ADHD should be researched to further understand the role and impact of this treatment modality. Case studies on multidisciplinary integrated therapeutic approaches to treating ADHD could further elucidate the process. Additional studies can explore NTs’ experiences in treating other neurodevelopmental disorders.

## Conclusion

Neurofeedback therapists interviewed in this study experienced neurofeedback as an effective and valuable treatment of ADHD to reduce symptom severity and improve the patient’s quality of life, particularly when used as part of a multimodal approach. Common comorbidities of ADHD patients such as anxiety, depression and insomnia were also reported to improve with the use of this therapy. Neurofeedback therapists were aware of the limitations of neurofeedback and expressed frustration with the challenges they faced, such as poor patient compliance and feedback and misconceptions about neurofeedback. A positive therapeutic relationship between the patient and NT was experienced as a vital cornerstone in improving therapeutic outcomes, and the need for further education and awareness regarding the use of neurofeedback for ADHD was identified. This study has contributed to an understanding of the role of neurofeedback in the treatment of ADHD in the South African setting.
